# Estimating the 15th‐Century Potential Habitats of Endangered Mammals on the Korean Peninsula: Implications for Restoration

**DOI:** 10.1002/ece3.71676

**Published:** 2025-06-27

**Authors:** Dabin Kim, Kyung Ah. Koo

**Affiliations:** ^1^ Korea Institute of S&T Evaluation and Planning Eumseong‐gun Chungcheongbuk‐do Republic of Korea; ^2^ Korea Environment Institute Sejong Republic of Korea

**Keywords:** endangered mammals, historical document, mapping past distributions, the Korean peninsula, tribute records

## Abstract

Understanding the past distribution is valuable reference information for restoring endangered species that lack current suitable habitat information. We, thus, estimated the 15th‐century potential habitats of critically endangered mammals at the genus level on the Korean Peninsula with a historical document: big cats (*Panthera* spp.), including tigers and leopards, foxes (*Vulpes* spp.), bears (*Ursus* spp.), and gorals (*Naemorhedus* spp.). For this, we mapped the district‐based mammals' habitats using the tribute records of local mammalian products in Sejong Silok jiriji, a historical document written in the 1400s on a peninsula scale. The habitats of all mammal genera mainly included the Baekdudaegan mountain range, stretching from North to South Korea, and were commonly found in the two provinces of North Korea: Hamgyung‐do and Pyungan‐do. Especially, foxes showed the peninsula‐wide habitat distribution, including non‐forest areas. The common characteristics of their habitats were high‐altitude mountainous areas with steep slopes and rugged topography. Contrary to the current limited ranges of the corresponding mammal species on the Korean Peninsula, especially in South Korea, the 1400s estimations showed peninsula‐wide distributions of the four mammal genera. Despite several limitations of historical documents, such as presence‐only administrative and genus‐level information, estimating mammals' habitats using historical records is a novel and important approach, highlighting the value of these records in understanding past mammals' habitat distributions and characteristics. Our results provide valuable reference information for the restoration and conservation practices of the four critically endangered mammals, with limited knowledge of their suitable habitat conditions in the Republic of Korea.

## Graphical abstract

We estimated the 15th‐century potential habitats of endangered mammals at the genus level on the Korean Peninsula with a historical document: big cats (Panthera spp.), including tigers and leopards, foxes (Vulpes spp.), bears (Ursus spp.), and gorals (Naemorhedus spp.). Estimating mammals' habitats using historical records is a novel and important approach; our results provide valuable reference information for the restoration of the four endangered mammals. Despite several limitations of historical documents, such as administrative and genus‐level information, our results highlight the value of these records in understanding past mammals' habitat distributions and characteristics.

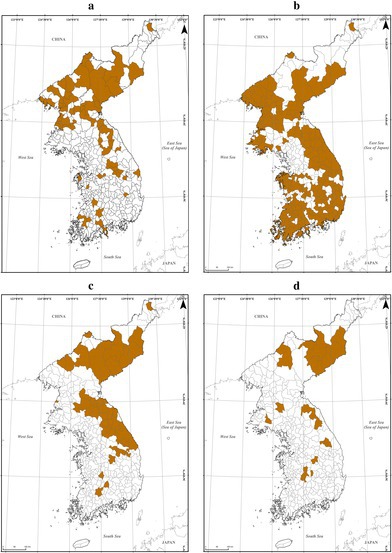

## 1 Introduction

Understanding the past distribution of animal species is fundamental in zoogeography, biogeography, and historical ecology (Lomolino and Channell 1995; Sanderson et al. 2002; Fjeldså et al. 2004). The distributions of animal species provide various information regarding their socioecological habitat conditions, including climate conditions, land covers, topographical characteristics, and human activities such as poaching, etc. Thus, the reconstruction of the past animal distributions helps us to estimate their suitable habitat conditions, such as suitable temperature and precipitation ranges, land‐cover types, etc., and understand the socioecological mechanism of spatiotemporal changes in distributions (Kerley et al. 2003; Kim 2017a; Kong et al. 2016; Kim et al. 2019). Such information is especially valuable for restoring endangered species lacking suitable habitat information, as it provides a crucial foundation for identifying habitats for restoration (Kim et al. 2003; Boshoff and Kerley 2010; Choi and Park 2004).

Historical ecology and zoogeography have frequently used proxy data, including fossils, pollen, cave paintings, and historical documents, to estimate a species' past distributions (Kong 1996; Cho 2014; Boshoff and Kerley 2010; Kong et al. 2014). The records of historical documents, which reflect environmental conditions when the documents were created, have been used to reconstruct the mammals' distribution in association with environmental conditions (Boshoff and Kerley 2010; Zhang et al. 2016; Salari et al. 2020). The distribution of the Eurasian otter ( *Lutra lutra* ) was reconstructed in northeastern China from 1950 to 2014 by analyzing papers published in the 1960s and local historical documents, such as official gazettes, chronicles, and journals (Zhang et al. 2016). The distribution of the Italian European beaver ( *Castor fiber* , Eurasian beaver) was reconstructed using several proxy data sources, such as fossils and historical documents, from the late Pleistocene era to the 16th and 17th centuries in association with environmental changes and anthropogenic disturbances, such as overhunting and habitat loss (Salari et al. 2020).

There are 95–125 mammalian species that are known to inhabit the Korean Peninsula, about 95 and 125 mammalian species inhabit the Democratic People's Republic of Korea (DPRK, hereinafter referred to as North Korea) and the Republic of Korea (ROK, hereinafter referred to as South Korea), respectively (NIBR 2012). However, many mammals have become locally extinct or critically endangered due to habitat loss attributed to urbanization, forest destruction, expanding agricultural areas, industrialization, and climate change (So et al. 2016). South Korea needs information on the suitable habitat conditions for restoring endangered species to recover the biodiversity of Korea, but very limited information is currently available, especially for endangered mammal species. This information would be obtained from the estimations of past distribution (Kim et al. 2020, 2019; Boshoff and Kerley 2010).

The Republic of Korea (ROK) has well‐developed historical documents reflecting local specialties, such as natural resources and mammalian products like leather, skin, meat, fur, etc. Previous studies have reported the past distribution of mammals reconstructed from historical documents at a local scale (Jung et al. 2016; Kim et al. 2019, 2020). Those studies provide valuable information to study and understand the species' past habitat conditions. However, despite the usefulness, those studies have only focused on specific species or regions, limiting the understanding of the habitat characteristics of mammals on the scale of the Korean peninsula. Therefore, we need the peninsula scale studies to estimate various mammals' past distributions, which provides a better understanding of their suitable habitat conditions and ranges.

In this study, we aim to (1) provide the reference information of suitable habitat conditions of the four critically endangered mammals at the genus level by reconstructing their past habitat distributions in the 1400s at the peninsula scale using historical data and (2) explain declines of their range by comparing the current and the 1400s distributions. For this, we estimate and map the past distributions of key endangered mammals' habitats on the Korean Peninsula using the records of the Geography Section of the Annals of King Sejong, called Sejong Sillok Jiriji (1425–1454) and compare the estimated distributions with the current distributions of the corresponding species studied by the National Institute of Biological Resources (NIBR 2018). Our results will provide information about potential historic distributions of the four mammal genera, which is valuable information on the conservation and restoration of those endangered mammals with limited knowledge of their suitable habitats in South Korea.

## 2 Materials and Methods

### 2.1 Study Mammals and Site

The study mammals are big cats (tigers and leopards), foxes, bears, and gorals at the genus level. The corresponding species belonging to these mammals' genera that currently inhabit the Korean Peninsula are the Siberian tiger ( *Panthera tigris altaica* ) and the Amur leopard ( *Panthera pardus orientalis* ) for big cats, the Korean fox ( *Vulpes vulpes peculiosa* ) for foxes, the Ussuri black bear ( *Ursus thibetanus ussuricus* ) for bears, and the Long‐tailed goral ( *Naemorhedus caudatus* ) for gorals, all of which are critically endangered mammals in South Korea (So et al. 2016). Among them, the Siberian tiger and the Amur leopard are locally extinct, and the Korean fox, the Ussuri black bear, and the Long‐tailed goral are under the national restoration projects (https://www.knps.or.kr/portal/main/contents.do?menuNo=7020028).

In general, the historical record could have limitations in taxonomically matching them at the species level due to the absence of the current taxonomic system in the past (Boshoff and Kerley 2010). We also do not have any other historical and scientific data collected in the Chosun Dynasty to enable us to make the species‐level identification. However, the mammals' by‐products in the Jiriji represented typical characteristics of each mammal could distinguish them at the genus level. The genus level data could provide limited information on habitat suitability for the specific species but still very useful in estimating potentially suitable habitats for a species with very limited ecological data and knowledge as reference data, because species belonging to the same genus with morphological similarities could have similar suitable habitat conditions and ecological functions in the ecosystem (Burns and Strauss 2011). So, providing information on the mammal's habitats at the genus level is still valuable as reference data for restoring the endangered mammals with the peninsula‐wide territories in situations of limited current data and information, especially little official information on those mammals in North Korea.

**FIGURE 1:**
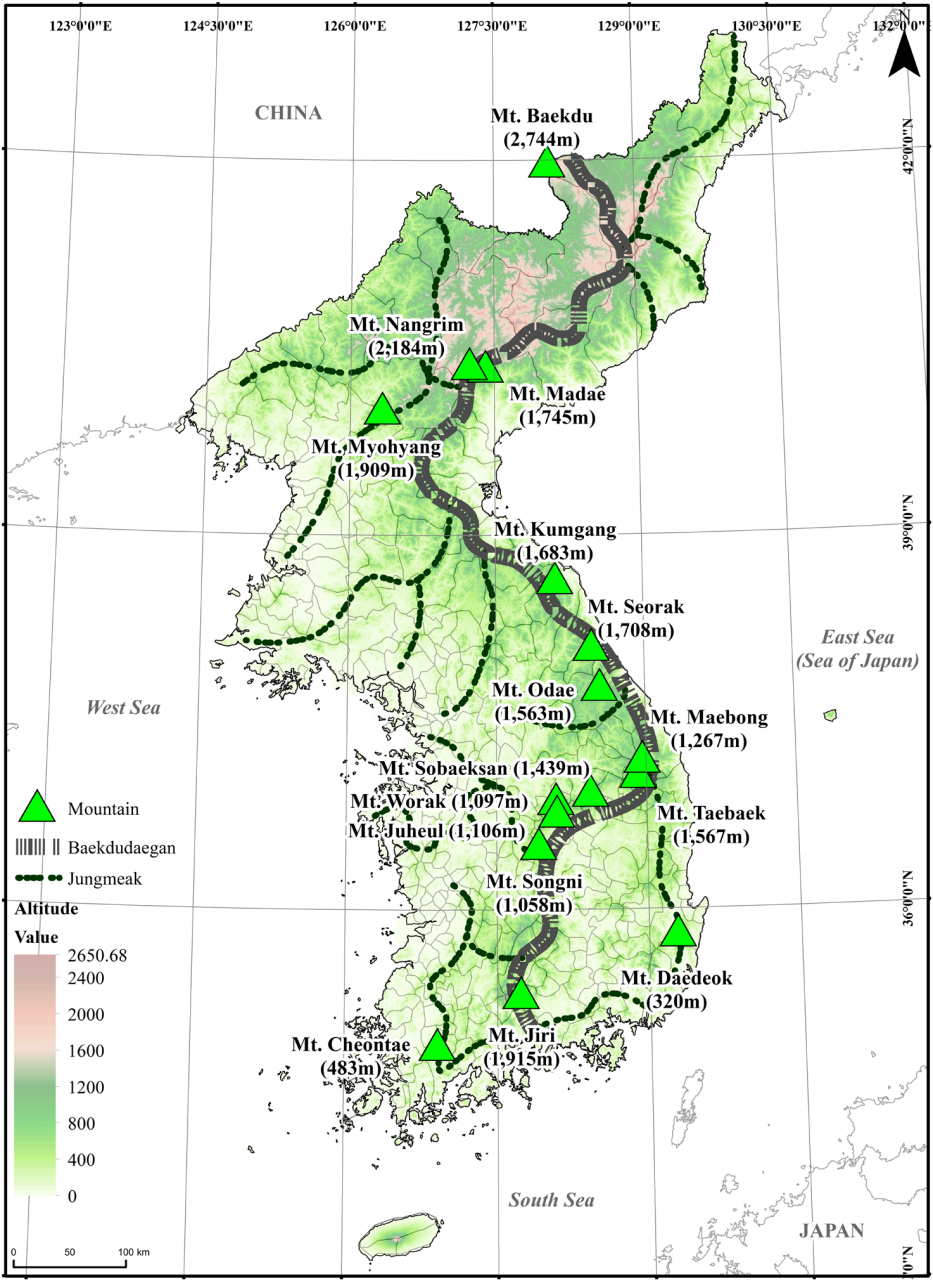
The study site, the Korean Peninsula (KP): The map shows the geographical location, topographical characteristics, and the mountain chain, Baekdudaegan mountain range, of KP. The Baekdudaegan mountain range includes major mountains and causes the topographical east‐high and west‐low characteristics in KP.

The study area was the Korean Peninsula (KP) (Figure 1), in the eastern part of the Eurasian continent. The northernmost point of the Korean Peninsula is located at 43°0′39″ N and the southernmost point at 34°17′21″ N. The Peninsula runs straight from north to south at about 1013 km; it has various regional climatic conditions (Figure 1) (Oh 2004). The southern part, including Jeju‐do and below, has a subtropical climate, while the middle part has a temperate climate. The climate of the northern peninsula is mostly classified as a cold climate, −3°C ~ −13°C in January and 29°C ~ 20°C in August of the daily average temperature range (Pyongyang) [https://archive.md/20121212003435/http://lcweb2.loc.gov/cgi‐bin/query/r?frd/cstdy:@field(DOCID+kp0031)], while the northeastern part, including the Gaema Plateau and some subalpine regions, shows a boreal climate in which the temperature is cool in summer and severely cold in winter (Oh 2004). The Peninsula has about 67% mountainous areas with a high mountain linear range called Baekdudaegan, extending from Mt. Baekdu (2744 m) in the north to Mt. Jiri (1915 m) in the south (Kwon et al. 2016). The Baekdudaegan forms the east‐high–west‐low landscape of the Peninsula (Figure 1) (Oh 2004). Such landscapes and climatic conditions have shaped various habitats for mammals.

### 2.2 Data Analysis and Spatialization

**FIGURE 2:**
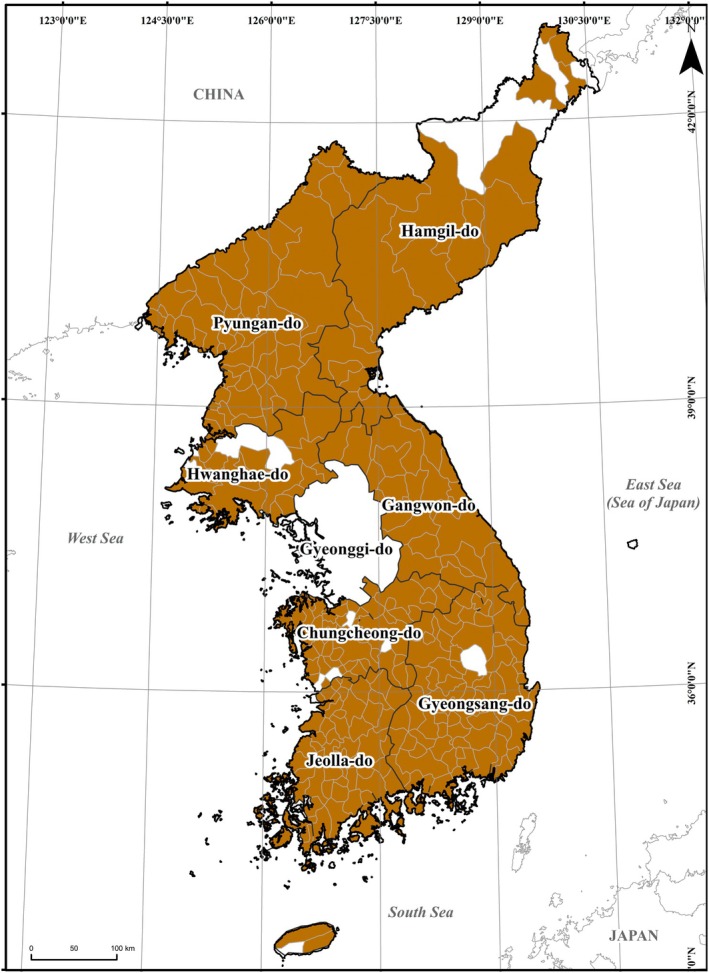
The eight administrative provinces and the 275 districts with mammal‐related tributes of the Korean Peninsula in the 1400s.

We analyzed Sejong Sillok Jiriji, written in the 15th century, 1425–1454, to estimate the historical distribution of the four endangered mammals based on the administrative spatial unit. It is in the national geography section of the Annals of King Sejong, called Sejong Jangheon Daewang Sillok, with volumes from 148 to 155 (https://sillok.history.go.kr/id/kda_400). This document contained various local information, such as population, land area, local specialties, tributes, etc. The tributes were local governments' taxes to the federal government, the royal court at the time. The federal government documented many types of locally produced tribute in the geography section, including local crops, medicinal herbs, animal products, etc., in the district units, such as Bu, Mok, Dohobu, Gun, and Hyun, for all eight provinces (Figure 2, Supporting Information: Table A1) (https://sillok.history.go.kr/id/kda_400). The eight provinces, including Hamgil‐do, Pyungan‐do, Hwanghae‐do, Gyeonggi‐do, Gangwon‐do, Chungcheong‐do, Gyeongsang‐do, and Jeolla‐do, were reconstructed based on the records of the Jiriji. Those records precisely represented the district unit‐based distribution of tributes due to a strict recording rule, which recorded the tributes captured at the corresponding district (Kim 1987; Lee 1998). Particularly, we here focused on the information on mammal tributes of the four mammal genera in estimating their distributions in the 1400s (Kim et al. 2020), which is related to the corresponding critically endangered species in South Korea.

We first identified the tributes related to the four target mammal genera in the Jiriji, listed the tributes for each mammal genus, mapped districts with the corresponding lists, and finally reconstructed the 15th‐century distributions of four mammals' habitats at the genus level at the peninsular scale. The mammal‐related tributes were mammals themselves, leather, bones, organs, hair, horns, tail, and meat, which were used for medicinal materials, food, clothing, and ornaments. For example, tributes related to bears include bear, bear gallbladder, bear hair, bear leather, etc. The distributions of mammals' habitats were mapped based on the district unit using ArcMap 10.3. For the mapping, we first obtained the shapefile of the administrative map of the Chosun Dynasty from the Chosun Culture Electric Atlas of Research Institute of Korean Studies, Korea University (http://www.atlaskorea.org/historymap.web/IdxRoot.do). Each district has a unique identification (ID) code. Second, we created an attribute table for each mammal genus, including the district ID and a binary variable, indicating each district's presence (1) or absence (0) of tribute based on the records of the Sejong Sillok Jiriji. Third, we incorporated the administrative map and the attribute table based on the common district ID using the ‘Join’ tool in ArcMap 10.3. Finally, the district with tribute records was selected using the ‘Definition Query’ tool to generate the tribute‐based distribution map of each mammal genus.

We matched the four mammal genera with the corresponding current mammal species based on the prior studies that listed and reported the mammals in the Sejong Sillok Jiriji (Yu 2000; Jung et al. 2016). Then, we compared the reconstructed habitat distributions with the present distributions of the corresponding species to explain the changes in distribution since the 1400s. The present distributions of those species were identified from the report of the National Institute of Biological Resources (NIBR), “Endangered Wildlife at a Glance,” published in 2018 (NIBR 2018).

## 3 Results

### 3.1 Tributes of the Four Mammal Genera in the Sejong Sillok Jiriji

The Jiriji recorded 82 types of mammal‐related tributes, such as mammals themselves, leather, fur, oil, bones, organs, etc., and all mammals were recorded as more than two types of tributes (Table 1). Big cats have five tributes: tiger, tiger leather, tiger tibia bone, leopard tail, and leopard leather; foxes have three tributes: fox, fox leather, and fox tail; bears have four tributes: bear, bear leather, bear gallbladder, and bear hair; and gorals have five tributes: antelope, goral, antelope horn, goral horn, and goral leather. The tributes of mammals were distinguished by their unique name written in Chinese letters.

**TABLE 1:** The records for the tributes of four mammal genera identified in the Sejong Sillok Jiriji.

Order	Mammals	Records
	Big cats	Leopard tail (豹尾), leopard leather (豹皮), tiger (虎), tiger leather (虎皮), and tiger tibia bone (虎脛骨)
	Foxes	Fox (狐), fox leather (狐皮), and fox tail (狐尾)
	Bears	Bear (熊), bear leather (熊皮), bear gallbladder (熊膽), and bear hair (熊毛)
Artiodactyla (偶蹄目)	Gorals	Antelope (羚羊), long‐tailed goral(山羊), antelope horn (羚羊角), antelope horn (羚羊角), long‐tailed goralhorn (羚羊角), long‐tailed goralleather (阿羊鹿皮), and long‐tailed goralhorn (阿羊鹿角)

*Note:* The Chinese letter for each tribute is presented in parentheses. The Chosun Dynasty collected the tributes according to the fundamental and strict rule. The rule is that mammal‐based tributes had to be produced within the district using resources sourced from that same district. Furthermore, the name of each tribute indicated the origin of the mammal by‐product, specifying the mammal from which it came.

### 3.2 Districts Recorded the Four Mammals' Tributes in the Sejong Sillok Jiriji

Out of the 334 districts, 275 had records of mammal tributes in the 1400s (Figure 2). No record of mammal tributes was found in Gyeonggi‐do, while Pyungan‐do and Gangwon‐do presented the tributes' records in 47 and 24 districts, respectively. Jeolla‐do showed the records in 54 of 56 districts, Hamgil‐do in 17 of 20, Gyeongsang‐do in 63 of 66, Hwanghae‐do in 19 of 23, and Chungcheong‐do in 51 of 55.

The tributes of foxes were recorded in more than 200 districts. The tributes of big cats, bears, and gorals were recorded in 63, 43, 22, and 18 districts, respectively (Table 2, Supporting Information: Tables A2–A5). The results of the analyses showed that the big cat‐related tributes were mostly observed in Pyungan‐do (32 districts), the fox‐related tributes in Gyeongsang‐do (44 districts), and the bear‐related tributes in Gangwon‐do (16 districts). The goral‐related tributes were mostly found in five districts of Hamgil‐do.

**TABLE 2:** The number of districts in each province with records of tributes related to the four mammals in the Sejong Sillok Jiriji (1400s). The tributes of big cats are mostly found in Pyungan‐do, foxes in Gyeongsang‐do, bears in Gangwon‐do, and gorals in Hamgil‐do.

Mammal group	Provinces							Total
	Hamgil‐do	Pyungan‐do	Hwanhae‐do	Gangwon‐do	Chungcheong‐do	Gyeongsang‐do	Jeolla‐do	
Big cats	9	32	—	6	8	2	6	63
Foxes	11	39	8	22	33	44	43	200
Bears	9	6	2	16	4	4	2	43
Gorals	6	1	2	4	2	1	2	18

### 3.3 Distributions of the Four Mammals' Habitats in the 1400s

**FIGURE 3:**
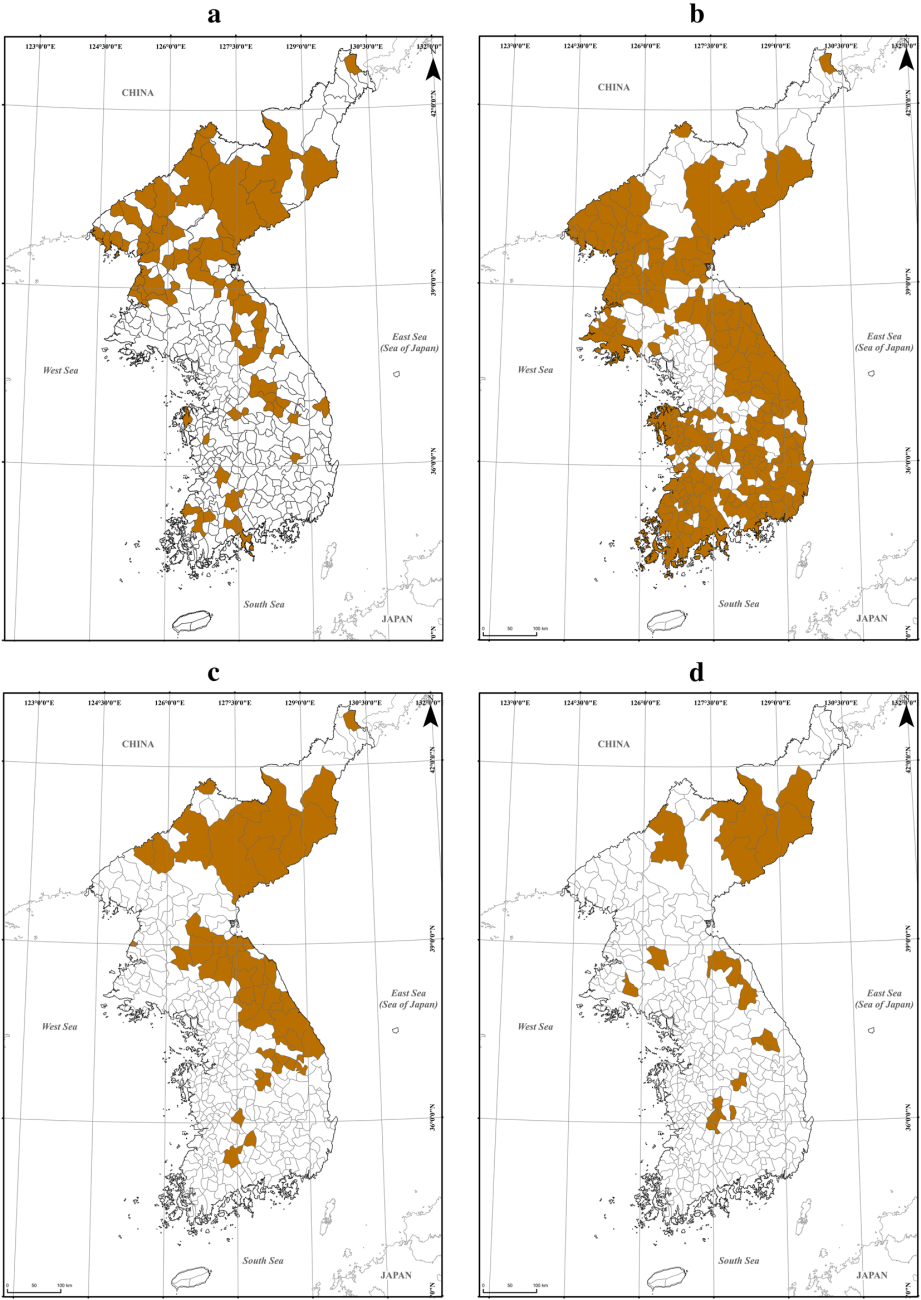
The distribution maps of four mammal groups in the 1400s. The maps show the districts with the tribute's records of the four groups, including big cats (a), foxes (b), bears (c), and gorals (d).

The tributes of big cats were mostly recorded in the 63 districts with high and rugged mountains, approximately over 1400 m above sea level altitude (Table 2, Supporting Information: Table A2, Figure 3a). Those mountains were Mt. Baekdu in Hamgil‐do (2750 m), Mt. Myohyang in Pyungan‐do (1909 m), Mt. Kumgang in Gangwon‐do (1638 m), Mt. Sobaek in Gyeongsang‐do and Chungcheong‐do (1439 m), and Mt. Jiri (1814 m) in Jeolla‐do and Gyeongsang‐do (Figure 3a, Figure 1). The tributes of the foxes were offered to the federal government in most areas except Gyeonggi‐do and the northern part of Pyungan‐do and Hamgil‐do, including 200 districts of the Korean Peninsula (Table 2, Supporting Information: Table A3, Figure 3b). The tributes of bears were mostly found in the districts of Hamgil‐do and Gangwon‐do (Table 2, Supporting Information: Table A4, Figure 3c). These districts were along the Baekdudaegan linear mountain range and other small mountain ranges. The bear tributes were found in almost all high mountains of the Peninsula, from Hamgil‐do, the northernmost province, to Jeolla‐do, the southernmost province. The records of gorals' tributes were found in parts of Gangwon‐do, eastern Chungcheong‐do, and northeastern Jeolla‐do along the continuous mountainous linear range of Baekdudaegan (Table 2, Supporting Information: Table A5, Figure 3d).

### 3.4 Declines in the Distributions of Four Mammals Since the 1400s

Approximately 50 Siberian tigers ( *P. tigris altaica* ), one species of the big cats, are currently distributed in northeastern China, Manchuria, Jilin Province, Songhua River, Mudan River, Ussuri, and northeastern Russia (NIBR 2018). A few Siberian tigers have been observed in Hamgyung‐do (the current name of Hamgil‐do) and Pyungan‐do, North Korea (NIBR 2018). The Amur leopard ( *P. pardus orientalis* ), the other species of the big cats, currently lives in Manchuria, the far east Primorsky Krai and Amur River area in Russia, and northern China (NIBR 2018). In the Korean peninsula, leopards inhabited mountains in North Korea, such as Mt. Myohang and Mt. Baekdu, in Pyeongan‐do and Hamgyung‐do, and mountains in South Korea, including Mt. Jiri, Mt. Cheontae, Mt. Seorak, and Mt. Odae, in Gangwon‐do, Chungcheong‐do, and Jola‐do during the 1400s. Compared with the distribution in the 1400s, the big cats have lost most of their habitats in North Korea and all their habitats in South Korea. The population of big cats rapidly declined, and they lost all their habitats, finally leading to their extinction in South Korea (NIBR 2018).

The Korean fox ( *V. vulpes* ) is widely distributed in the northern hemisphere, from the Eurasian continent, North Africa, Canada, and the United States of America to the Korean Peninsula (NIBR 2018). The records of fox tributes in the Jiriji were shown to have a nationwide distribution in the 1400s (Figure 3b). The foxes were distributed throughout the Korean Peninsula and were frequently observed near mountains, forests, grasslands, and even villages until the 1960s (NIBR 2018). The foxes are known to inhabit North Korea; however, wild Korean foxes in South Korea were reported extinct in 1992 (NIBR 2018). A few Korean foxes have been reintroduced into the wild through national restoration projects. These reintroduced individuals are the only confirmed wild Korean foxes currently identified in South Korea.

The Ussuri black bear ( *U. thibetanus ussuricus* ) is distributed in the Korean Peninsula, northeastern China, and the Maritime Province of Russia (NIBR 2018). Several Ussuri black bears probably inhabit North Korea; however, we cannot grasp accurate information on their populations and habitats due to political reasons. While our results showed that the bears were distributed in almost all high mountains of the Peninsula, from Hamgil‐do, the northernmost province, to Jeolla‐do, the southernmost province in the 1400s (Figure 3c), the bears lost most of their habitats and became an endangered species in South Korea. The restoration project of the Ussuri black bear has continued in Mt. Jiri, Jeolla‐do, the southernmost part of Baekdudaegan, since 2004, and small numbers of the Ussuri black bear were found through the national restoration project.

Long‐tailed goral ( *N. caudatus* ), mainly distributed in the Korean Peninsula, is also observed in northeastern China, Amur, Ussuri, and Heilong Jiang basin forest areas (NIBR 2018). The gorals were commonly observed in parts of Gangwon‐do, eastern Chungcheong‐do, and northeastern Jeolla‐do along the continuous mountainous linear range of Baekdudaegan in the 1400s (Figure 3d). However, long‐tailed gorals are currently distributed in rugged mountainous areas of the Baekdudaegan and the DMZ in South Korea, including Mt. Juheul in Gyeongsang‐do, Mt. Worak in Chungcheong‐do, Mt. Taebaek, Mt. Odae, Mt. Seorak, and the Daegwanryeong plateau in Gangwon‐do (NIBR 2018). Compared with the records from the 1400s the habitats of gorals in the middle and southern parts of South Korea have been lost and only remain in the northeastern areas, including mountainous areas in Gangwon‐do and the DMZ.

## 4 Discussion

For the four mammal genera, big cats (Panthera spp.), including tigers and leopards, foxes (Vulpes spp.), bears (Ursus spp.), and gorals (Naemorhedus spp.), we mapped the district‐based mammals' habitats in the 1400s on a peninsula scale. The habitats of the four mammal genera were mainly distributed in the Baekdudaegan mountain range and were commonly found in Hamgyung‐do and Pyungan‐do of the northeastern Peninsula. The common characteristics of their habitats were high‐altitude mountainous areas with steep slopes and rugged topography. The Siberian tiger ( *P. tigris altaica* ), Amur leopard ( *P. pardus orientalis* ), Korean fox ( *V. vulpes peculiosa* ), Ussuri black bear ( *U. thibetanus ussuricus* ), and long‐tailed goral ( *N. caudatus* ) are locally extinct or critically endangered on the Korean Peninsula, particularly in South Korea. However, although identified at the genus level, the estimations indicate the peninsula‐wide distributions of these in the 1400s. The reasons for the extinction or endangerment of big cats, foxes, gorals, and bears in South Korea have not been sufficiently studied, but habitat destruction might be a major reason for their extinction and drastic declines (NIBR 2018). The Korean War and the extensive developments of urban, roads, industrial, and agricultural lands according to the rapid economic growth of South Korea since the 1960s have led to massive habitat losses (Brady 2008). In particular, the populations and habitats of big cats (tigers and leopards) rapidly declined due to the increased demand for fur, excessive hunting using rifles (since the late 16th century), and habitat destruction, leading to extinction in South Korea (NIBR 2018). Tigers in China have also shown a rapid population decline and habitat loss (Kang et al. 2010). Tigers have entirely vanished from the temperate broadleaf and mixed forests of central China, and the survival of wild tigers in China now heavily relies on substantial conservation measures (Kang et al. 2010).

According to the international and domestic demand for endangered wildlife conservation and restoration (Bangs 1996; Beier et al. 2003; Breitenmoser et al. 1999; Ripple and Beschta 2004), all endangered animal species' habitats have been protected by strong conservation laws, such as Wildlife Protection and Management Act (https://elaw.klri.re.kr/kor_service/lawView.do?hseq=24747&lang=ENG), Conservation and Management of Marine Ecosystem Act. (https://elaw.klri.re.kr/kor_service/lawView.do?hseq=55797&lang=ENG), and Cultural Heritage Protection Act. (https://elaw.klri.re.kr/kor_service/lawView.do?hseq=67730&lang=ENG), as protected areas in South Korea. Human activities, which cause habitat degradation and loss, are strongly restricted in these mammals' habitats. In addition to this, the Ussuri black bear, long‐tailed goral, and Korean fox have been introduced in South Korea (Kang and Paek 2005; Park et al. 2008; Cho et al. 2013, 2014). Most projects and studies of endangered wildlife restoration have mainly been based on analyses of the habitat and ecological characteristics of each species at relatively small spatial scales, such as local and provincial scales (Kang and Paek 2005, Park et al. 2008, Cho et al. 2013, 2014). However, considering the wide ranges of their territories reflected in the peninsula‐wide distributions of big cats, bears, and foxes' habitats in the 1400s, it is necessary to analyze the characteristics of those mammals' habitats at the peninsula scale for supporting the continuous restoration projects of bears and foxes and the future projects for the big cats.

In the 1400s, the big cats and bears were commonly distributed across the mountainous terrain of the Baekdudaegan mountain range, which extends continuously from north to south on the Korean Peninsula and spans multiple climatic zones (Figure 1). The records in the 1400s showed the big cats and bears were found in Mt. Myohyang in North Korea, Mt. Jiri and Mt. Cheontae in Jeolla‐do province, and Mt. Seorak and Mt. Odae in Gangwon‐do province. Considering the wide ranges and continuity of bears and big cats' habitats in the 1400s, habitat connectivity is a crucial factor for their restoration. Thus, the whole area of the Baekdudaegan mountain range, the key habitats for bears and big cats, better be continuously protected by strong conservation laws, and the damaged or destroyed areas on the Baekdudaegan should be restored. The gorals inhabited parts of Gangwon‐do, eastern Chungcheong‐do, and northeastern Jeolla‐do along the continuous mountainous range of Baekdudaegan in the 1400s (Figure 3d). However, they are currently found in the rugged, high‐elevation mountainous areas of the Baekdudaegan, where humans and other animals have limited access, specifically in Gangwon‐do, Chungcheong‐do, and the DMZ (NIBR 2018). Such characteristics of gorals' habitats imply that the gorals are very vulnerable to human disturbances. Thus, we need strong conservation laws restricting human activities to protect the goral habitats. The records of fox tributes presented the nationwide distribution of foxes in the 1400s (Figure 3b), and the foxes inhabited near mountains, forests, grasslands, and even villages until the 1960s (NIBR 2018). Therefore, habitat protection is not necessary for the foxes, but rather proper restoration projects, such as captive breeding programs and reintroduction of individuals.

Such characteristics of the mammal genera's distributions in the 1400s provide the reference information to identify and select adequate habitats for their restorations. For example, the next restoration project of the bear is planned at Mt. Seorak in Gangwon‐do (https://www.knps.or.kr/portal/main/contents.do?menuNo=7020028), which is included in its distributional range of the 1400s. Big cats (tigers and leopards) are not restored in South Korea for various reasons, including habitat fragmentation (Chung 2022; Ministry of Environment 2006). Still, they can be restored at the high altitudes and rugged mountainous areas of the Baekdudaegan mountain range in South Korea under the restoration of connectivity among mountains. Based on estimates of their habitats in the 1400s, gorals could be restored to high‐elevation, rugged mountainous areas in northeastern Jeolla‐do. Based on the habitats' distributions and characteristics in the 1400s, foxes can be easily restored in most habitat types under current environmental conditions, but research on potential fox‐human conflicts is needed before restoration.

Overall, the South Korean government has protected and restored the habitats of those critically endangered mammals at the national scale of South Korea through strong conservation laws and policies. The government has made significant progress, but many gaps remain. Most gaps lie in the lack of science‐ and evidence‐based policymaking specialized for each mammal species, secure long‐term financing, and multilevel cooperative governance involving private and public stakeholders. Securing long‐term funding to implement diverse and sustained socio‐ecological research projects for each mammal species is essential for species‐specific policymaking and effective conservation and management practices. Establishing multilevel cooperative governance at local, national, regional, and global scales is crucial for mammals with cross‐boundary ranges, including those spanning state and national borders. In particular, regional cooperation with North Korea, China, and Russia is crucial for the restoration of big cats and bears, which require connected habitats across the peninsula and northeast Asia to sustain viable populations.

Estimating the mammals' habitats at the genus level using massive, detailed, and well‐organized historical documents at a national scale is globally novel. It would be an important attempt for further paleoenvironmental studies. No previous study had used the recorded detailed information based on local products made with living organisms collected from 330 administrative districts (Kim 2009, 2017b). However, the historical document “Sejong Sillok Jiriji” was published for administrative purposes of the federal government rather than scientific research. Thus, Jiriji has several limitations in interpreting the results. First, despite the strict recording rule, a few records probably include local specialties and products not produced in the district, which limits the estimation of the exact location of mammals' habitats. Second, as with other historic data, presence‐only data, the absence in the Jiriji data does not indicate true absence but potential absence, which increases the uncertainty in estimating past distributions. Third, the animal classification system of the Joseon Dynasty did not follow the modern taxonomic system, restricting the accurate matching of the mammals in the 1400s with the present ones at the species level. However, despite these limitations, historical documents are useful in estimating the geographical locations of animal habitats in the past at the genus level with morphological similarities. This is valuable reference information for understanding the animals' habitat conditions and ranges and estimating related changes in the ecosystem and environment, especially for critically endangered species with minimal ecological knowledge.

## Author Contributions

**Dabin Kim:** conceptualization (equal), data curation (lead), formal analysis (lead), investigation (lead), methodology (equal), project administration (supporting), supervision (supporting), validation (supporting), visualization (lead), writing – original draft (supporting), writing – review and editing (supporting). **Kyung Ah. Koo:** conceptualization (equal), data curation (supporting), funding acquisition (lead), methodology (equal), project administration (lead), resources (supporting), software (supporting), supervision (lead), writing – original draft (equal), writing – review and editing (lead).

## Conflicts of Interest

The authors declare no conflicts of interest.

## Supporting information


Tables A1–A5.

## Data Availability

The data supporting the findings of this study are available on Figshare (https://doi.org/10.6084/m9.figshare.29149451) and from Kyung Ah Koo upon reasonable request. The datasets include the map of eight administrative provinces of the Korean Peninsula (Figure 2) and the map of 275 districts with mammal‐related tributes in the 1400s (Figure 2) identified from the Sejong Sillok Jiriji, the number of districts and names of districts for each province (Supporting Information: Table A1), tribute‐based habitat maps (Figure 3), and the number of districts and the names of districts in each province of each mammal (Supporting Information: Tables A2–A5). All GIS maps and data presented in this study will be made available on Figshare (https://doi.org/10.6084/m9.figshare.29149451) and from Kyung Ah Koo upon reasonable request. Access to these datasets will facilitate further research and understanding of the impacts of environmental and land‐use changes on endangered mammal species.
