# Supplementary material for: Estimating the 15th‐Century Potential Habitats of Endangered Mammals on the Korean Peninsula: Implications for Restoration
Source: Ecol Evol. 2025 Jun 27;15(7):e71676. doi: 10.1002/ece3.71676 (PMC12203234; doi:10.1002/ece3.71676)
Supplement: Supplementary file 1 — Tables A1–A5. [file ECE3-15-e71676-s001.docx]

**Appendix I**

**Table A1**. The number of districts and names of districts for each province

| **Provinces**  **(# of Districts)** | **Bu (府), Mok (牧), Gun (郡), Hyeon (縣)** |
| --- | --- |
| **Gyeonggi-do (43)** | - Gapyeong, Ganghwa, Gaeseong, Goyang, Gwacheon, Gwangju, Gyodong, Gyoha, Geumcheon, Gimpo, Namyang, Majon, Bupyeong, Sakryeong, Suwon, Ansan, Anseong, Anhyeop, Yanggeun, Yangseong, Yangju, Yangji, Yangcheon, Yeoheung, Yeongcheon, Yeongpyeong, Yongin, Wonpyeong, Eumjuk, Icheon, Incheon, Imgwang, Imjin, Jangdan, Jeokseong, Jipyeong, Jinwi, Cheollyeong, Cheolwon, Tongjin, Pocheon, Hanseong, Haepung |
| **Chungcheong-do (55)** | - Gyeolseong, Gongju, Goesan, Nampo, Nisan, Danyang, Dangjin, Daehung, Deoksan, Myeoncheon, Mokcheon, Munui, Boryeong, Boeun, Buyeo, Biin, Seosan, Seocheon, Seokseong, Sinchang, Asan, Yeongi, Yeonsan, Yeonpung, Yeongdong, Yeongchun, Yesan, Okcheon, Onsu, Eunjin, Eumseong, Imcheon, Jeonui, Jeongsan, Jecheon, Juksan, Jiksan, Jinjam, Jincheon, Cheonan, Cheongsan, Cheongan, Cheongyang, Cheongju, Cheongpung, Chungju, Taean, Pyeongtaek, Hansan, Haemi, Hongsan, Hongju, Hwanggan, Hoedeok, Hoein |
| **Gyeongsang-do (66)** | - Gaeryeong, Geoje, Geochang, Gyeongsan, Gyeongju, Goryeong, Goseong, Gonnam, Gunwi, Gijang, Gicheon, Gimsan, Gimhae, Daegu, Dongnae, Mungyeong, Miryang, Bonghwa, Bian, Sacheon, Saneum, Samga, Sangju, Seonsan, Seongju, Sunheung, Sinryeong, Andong, Aneum, Yangsan, Eonyang, Yeongdeok, Yeongsan, Yeongil, Yeongcheon (榮川), Yeongcheon (永川), Yeonghae, Yaean, Yecheon, Yonggung, Ulsan, Uiryeong, Uiseong, Uiheung, Indong, Janggi, Jirye, Jinbo, Jinseong, Jinju, Jinhae, Changnyeong, Changwon, Cheongdo, Cheongsong, Cheongha, Chogye, Chilwon, Hadong, Hayang, Haman, Hamyang, Hamchang, Hapcheon, Hyeonpung, Heunghae |
| **Jeolla-do (56)** | - Gangjin, Gobu, Gosan, Gochang, Goheung, Gokseong, Gwangyang, Gurye, Geumgu, Geumsan, Gimje, Naju, Nakan, Namwon, Nampyeong, Neungseong, Damyang, Daejeong, Dongbok, Mangyeong, Muan, Mujang, Muju, Mujin, Boseong, Buan, Sunchang, Suncheon, Yeosan, Yeonggwang, Yeongam, Okhwa, Okgu, Yongdam, Yongan, Unbong, Iksan, Imsil, Impi, Jangseong, Jangsu, Jangheung, Jeonju, Jeongeup, Jeongui, Jeju, Jinsan, Jinan, Jinwon, Changpyeong, Taein, Hamyeol, Hampyeong, Haejin, Hwasun, Heungdeok |
| **Hwanghae-do (23)** | - Gangryeong, Gangeum, Goksan, Munhwa, Baekcheon, Bongsan, Seoheung, Songhwa, Suan, Sineun, Sincheon, Anak, Yeonan, Ongjin, Ubong, Eunyul, Jangyeon, Jaeryeong, Tosan, Pyeongsan, Pungcheon, Haeju, Hwangju |
| **Gangwon-do (24)** | - Ganseong, Gangneung, Goseong, Geumseong, Kimhwa, Nangcheon, Samcheok, Yanggu, Yangyang, Yeongwol, Uljin, Wonju, Icheon, Inje, Jeongseon, Chuncheon, Tongcheon, Pyeonggang, Pyeongchang, Pyeonghae, Hongcheon, Hoeyang, Hoengseong, Heupgok |
| **Pyeongan-do (47)** | - Gasan, Ganggye, Gangdong, Gangseo, Gaechon, Gwaksan, Deokcheon, Maengsan, Muchang, Bakcheon, Byeokdong, Sakju, Samdeung, Samhwa, Sangwon, Seoncheon, Seongcheon, Sucheon, Sukcheon, Sunan, Suncheon, Anju, Yangdeok, Yeoheon, Yeongbyeon, Yeongyu, Yonggang, Yongcheon, Uye, Unsan, Wiwon, Eunsan, Uiju, Isan, Insan, Jasan, Jaseong, Jeongnyeong, Jeongju, Junghwa, Jeungsan, Changseong, Cheolsan, Taecheon, Pyeongyang, Hamjong, Huicheon |
| **Hamgil-do (20)** | - Gapsan, Gyeongseong, Gyeongwon, Gyeongheung, Gowon, Gilju, Dancheon, Muncheon, Buryeong, Bukcheong, Samsu, Anbyeon, Yeongheung, Yewon, Onseong, Uicheon, Jeongpyeong, Jongseong, Hamheung, Hoeryeong |

**Table A2**. The number of districts and names of districts for each province that the big cats’ tributes identified from the Sejong Sillok Jiriji

| **Province (# of Districts)** | **Districts** |
| --- | --- |
| **Gyeonggi-do (0)** | - |
| **Chungcheong-do (8)** | Seosan, Eumseong, Jincheon, Cheongyang, Danyang, Yeongchun, Jecheon, Cheongpung |
| **Gyeongsang-do (2)** | Yeongcheon (榮川), Uiheung |
| **Jeolla-do (6)** | Naju, Namwon, Mujin, Jeonju, Suncheon, Yeonggwang |
| **Hwanghae-do (0)** | - |
| **Gangwon-do (6)** | Wonju, Chuncheon, Hoeyang, Kimhwa, Yanggu, Uljin |
| **Pyeongan-do (32)** | Gasan, Ganggye, Gangseo, Gwaksan, Maengsan, Sakju, Samdeung, Sangwon, Seoncheon, Sucheon, Suncheon, Anju, Yangdeok, Yeoheon, Yeongbyeon, Yeongyu, Yonggang, Yongcheon, Uye, Unsan, Eunsan, Isan, Jaseong, Junghwa, Jeungsan, Cheolsan, Taecheon, Pyeongyang, Hamjong, Bakcheon, Byeokdong, Jeongnyeong |
| **Hamgil-do (9)** | Gapsan, Gyeongwon, Gilju, Bukcheong, Anbyeon, Yeongheung, Yewon, Jeongpyeong, Hamheung |

**Table A3**. The number of districts and names of districts for each province that the foxes’ tributes identified from the Sejong Sillok Jiriji

| **Province (# of Districts)** | **Districts** |
| --- | --- |
| **Gyeonggi-do (0)** | - |
| **Chungcheong-do (33)** | Gyulseong, Gongju, Goesan, Dangjin, Daehung, Deoksan, Myeoncheon, Mokcheon, Munui, Boryeong, Buyeo, Biin, Seosan, Asan, Yeongi, Yeongdong, Yesan, Okcheon, Onsu, Eunjin, Eumseong, Jeonui, Jeongsan, Jecheon, Jiksan, Jinjam, Jincheon, Cheongyang, Taean, Haemi, Hongsan, Hongju, Hoedeok |
| **Gyeongsang-do (44)** | Gaeryeong, Gyeongju, Goseong, Gunwi, Gijang, Gicheon, Gimsan, Gimhae, Daegu, Dongnae, Miryang, Bonghwa, Saneum, Sangju, Seonsan, Seongju, Sunheung, Sinryeong, Andong, Yangsan, Yeongdeok, Yeongcheon (榮川), Yeongcheon (永川), Yecheon, Yonggung, Ulsan, Uiryeong, Indong, Janggi, Jinbo, Jinseong, Jinju, Jinhae, Changnyeong, Changwon, Cheongdo, Cheongsong, Cheongha, Chogye, Chilwon, Haman, Hamyang, Hapcheon, Heunghae |
| **Jeolla-do (43)** | Gangjin, Gobu, Gochang, Goheung, Gokseong, Gwangyang, Gurye, Geumgu, Geumsan, Gimje, Naju, Nakan, Namwon, Nampyeong, Neungseong, Damyang, Muan, Mujang, Mujin, Boseong, Buan, Sunchang, Suncheon, Yeosan, Yeonggwang, Yeongam, Okhwa, Okgu, Yongan, Iksan, Imsil, Impi, Jangseong, Jangsu, Jangheung, Jeonju, Jeongeup, Jinan, Changpyeong, Taein, Hampyeong, Haejin, Heungdeok |
| **Hwanghae-do (8)** | Gang-eum, Munhwa, Baekcheon, Songhwa, Suan, Sincheon, Jangyeon, Haeju |
| **Gangwon-do (22)** | Ganseong, Gangneung, Goseong, Geumseong, Kimhwa, Nangcheon, Samcheok, Yanggu, Yangyang, Yeongwol, Uljin, Wonju, Icheon, Inje, Jeongseon, Chuncheon, Pyeonggang, Pyeongchang, Pyeonghae, Hongcheon, Hoeyang, Hoengseong |
| **Pyeongan-do (39)** | Gasan, Gangdong, Gangseo, Gaechon, Gwaksan, Deokcheon, Bakcheon, Byeokdong, Sakju, Samdeung, Samhwa, Sangwon, Seoncheon, Seongcheon, Sucheon, Sukcheon, Sunan, Suncheon, Anju, Yangdeok, Yeoheon, Yeongbyeon, Yeongyu, Yonggang, Yongcheon, Unsan, Uiju, Isan, Insan, Jasan, Jeongnyeong, Jeongju, Junghwa, Jeungsan, Changseong, Cheolsan, Taecheon, Pyeongyang, Hamjong |
| **Hamgil-do (11)** | Gyeongwon, Gowon, Gilju, Dancheon, Muncheon, Bukcheong, Yeongheung, Yewon, Uicheon, Jeongpyeong, Hamheung |

**Table A4**. The number of districts and names of districts for each province that the bears’ tributes identified from the Sejong Sillok Jiriji

| **Province (# of Districts)** | **Districts** |
| --- | --- |
| **Gyeonggi-do (0)** | - |
| **Chungcheong-do (4)** | Danyang, Yeonpung, Yeongchun, Jecheon |
| **Gyeongsang-do (4)** | Mungyeong, Bonghwa, Sunheung, Aneum |
| **Jeolla-do (2)** | Geumsan, Namwon |
| **Hwanghae-do (2)** | Goksan, Suan |
| **Gangwon-do (16)** | Gangneung, Goseong, Geumseong, Nangcheon, Samcheok, Yanggu, Yangyang, Icheon, Inje, Jeongseon, Chuncheon, Pyeonggang, Pyeongchang, Hongcheon, Hoeyang, Heupgok |
| **Pyeongan-do (6)** | Ganggye, Byeokdong, Yangdeok, Yeoheon, Isan, Hamjong |
| **Hamgil-do (9)** | Gapsan, Gyeongseong, Gyeongwon, Gilju, Dancheon, Bukcheong, Samsu, Anbyeon, Hamheung |

**Table A5**. The number of districts and names of districts for each province that the gorals’ tributes identified from the Sejong Sillok Jiriji

| **Province (# of Districts)** | **Districts** |
| --- | --- |
| **Gyeonggi-do (0)** | - |
| **Chungcheong-do (2)** | Okcheon, Hwanggan |
| **Gyeongsang-do (1)** | Mungyeong |
| **Jeolla-do (2)** | Geumsan, Yongdam |
| **Hwanghae-do (2)** | Suan, Jaeryeong |
| **Gangwon-do (4)** | Inje, Jeongseon, Pyeongchang, Hoeyang |
| **Pyeongan-do (1)** | Ganggye |
| **Hamgil-do (6)** | Gapsan, Gyeongseong, Gilju, Dancheon, Bukcheong, Samsu |
